# Adrenal lesions in young adults: variability in radiology reporting and implications for clinical evaluation

**DOI:** 10.1007/s00261-026-05606-x

**Published:** 2026-06-19

**Authors:** Malak Itani, Sarah Jacob, Daniel R. Ludwig, Mark J. Hoegger, L. Michael Brunt, T. K. Pandian, Irina Bancos, Natalia Genere

**Affiliations:** 1 Mallinckrodt Institute of Radiology, Washington University in St. Louis, St Louis, USA; 2 Division of Endocrinology, Metabolism and Nutrition, Duke University Hospital, Durham, USA; 3 Department of Radiology, Mayo Clinic, Rochester, USA; 4 Section of Minimally Invasive Surgery, Department of Surgery, Washington University in St. Louis, St Louis, USA; 5 Department of Surgery, Wake Forest University, Winston-Salem, USA; 6 Division of Endocrinology, Diabetes, Metabolism and Nutrition, Mayo Clinic, Rochester, USA; 7 Division of Endocrinology, Diabetes, Bone and Mineral Disorders, Department of Medicine,, Henry Ford Health System, Detroit, USA

**Keywords:** Adrenal mass, adrenal incidentaloma, reporting, diagnosis, imaging, radiology

## Abstract

**Objective:**

To evaluate the consistency and clinical implications of radiology reporting for adrenal lesions detected on cross-sectional imaging in a younger adult population.

**Methods:**

This IRB-approved minimal risk retrospective study included 253 patients aged 18–40 years with adrenal lesions identified on cross-sectional imaging (CT, MRI, and PET/CT). Radiology reports were reviewed to determine lesion descriptors, inclusion of the adrenal lesion in the report impression, and management recommendations. Logistic regression was used to identify factors associated with inclusion of adrenal lesions in the impression.

**Results:**

Among 253 adrenal lesions, 194 (76.7%) were included in the report impression and 59 (23.3%) were omitted. Lesion size was the most frequently reported descriptor (83.7% of reports), whereas attenuation measurements were reported in only 14.3%. Overall, 57.9% of reports contained no management recommendation. Follow-up imaging was recommended in 39.2% of cases, hormonal evaluation in 2.8%, and endocrine referral in 0.8%. Inclusion of adrenal lesions in the impression was independently associated with larger lesion size (OR 1.46 per cm, *p* = 0.025) but was not associated with imaging context, prior malignancy, or mechanism of discovery. Among 162 incidental lesions in patients without prior malignancy or known genetic predisposition, 5 (3.1%) were non-metastatic pheochromocytomas, 115 (71.0%) were benign, and 42 (25.9%) remained non-characterized.

**Conclusion:**

Adrenal lesions identified in younger adults are frequently omitted from radiology impressions and often lack key descriptors or management recommendations. Reporting practices appear to be influenced primarily by lesion size rather than clinical context, suggesting opportunities to improve the consistency and clinical utility of adrenal lesion reporting, particularly in younger patients where standardized evaluation strategies are less well established.

**Supplementary Information:**

The online version contains supplementary material available at https://doi.org/10.1007/s00261-026-05606-x.

## Introduction

The detection of adrenal incidentalomas has increased substantially over the past several decades, largely driven by expanding use and improving resolution of cross-sectional imaging. Early CT studies from the 1980 s and 1990 s reported prevalence rates below 1–2% [1, 2], whereas contemporary radiologic series now identify adrenal incidentalomas in approximately 4–5% of abdominal CT or MRI examinations [3, 4]. These rates approach those historically observed in autopsy cohorts [5]. The rising frequency of incidentally detected adrenal nodules has increased the clinical importance of accurate imaging characterization, consistent reporting, and appropriate recommendations for biochemical evaluation, follow-up imaging, and specialty referral. The 2017 American College of Radiology White Paper [6] focuses on imaging follow-up rather than biochemical evaluation and endocrine referral. According to the widely used 2023 European Society of Endocrinology and European Network for the Study of Adrenal Tumors (ESE/ENSAT) guidelines, all adrenal incidentalomas, defined as ≥ 1 cm, should undergo biochemical evaluation for hormone excess [7]. Specifically, all adrenal masses should be evaluated for cortisol hypersecretion with a 1 mg dexamethasone suppression test; evaluation for hyperaldosteronism is warranted in those with hypertension and pheochromocytoma screening is recommended in those with lipid-poor masses. Additionally, ESE/ENSAT guidelines recommend urgent assessment of adrenal masses in young patients, particularly those < 40 years [7].

Radiology report language plays a central role in the downstream management of adrenal incidentalomas. The terminology used by radiologists and the degree of emphasis placed on a finding, particularly whether a lesion is included in the report impression, may strongly influence clinicians’ perception of risk and subsequent diagnostic actions [8]. Despite this, direct evidence linking specific radiology reporting practices to downstream clinical evaluation remains limited. Prior studies have shown that management of adrenal incidentalomas is frequently inconsistent with established guidelines, with many patients failing to undergo recommended biochemical or imaging evaluation and others receiving follow-up testing that does not align with guideline recommendations [9–11]. Interventions that introduce structure into radiology reports, including standardized templates or embedded decision-support algorithms, have been associated with improved adherence to recommended evaluation strategies [12, 13]. These observations highlight the importance of understanding current reporting patterns and their potential impact on clinical care.

Adrenal nodule prevalence increases with age and subsequently, most existing research on adrenal incidentalomas has focused on adults over 50 years old [14, 15]. Consequently, considerably less is known about the characteristics and management of adrenal nodules detected in younger adults. Available data in patients younger than 40 years are limited to small series, endocrine-focused cohorts, or mixed-age studies in which younger patients represent only a small subset of the population. In a relatively large epidemiological study, Ebbehoj et al. demonstrated that incidence rates of adrenal tumors are markedly lower in young adults aged 18–39 years (9/100,000) compared to individuals 40–64 years old (66/100,000) and those older than 65 years (142/100,000) [16]. This relative lack of age-specific data creates uncertainty regarding optimal imaging evaluation and management strategies in younger individuals, who may differ in both underlying pathology and clinical risk profile compared with older populations [7, 17].

The purpose of this study was to evaluate adrenal lesions detected on cross-sectional imaging in young adults aged 18–40 years, with particular focus on radiology reporting practices and their potential clinical implications. Specifically, we assessed the frequency with which adrenal lesions were included in radiology report impressions, the completeness of imaging descriptors, the presence of management recommendations, and associations between reporting practices and downstream clinical evaluation.

## Methods

### Study design and data sources

This retrospective cross-sectional study was approved by the Institutional Review Board. Study data were stored in REDCap (Research Electronic Data Capture) hosted at Washington University in St. Louis with additional details provided in the acknowledgments. A radiology information system (RIS) search was performed to identify cross-sectional abdominal imaging studies (CT, MRI, and FDG PET/CT) performed between January 1, 2016 and December 31, 2021 in patients aged 18–40 years. Searches were conducted using Montage (Nuance, Burlington, MA; version 2022) and included the terms “adrenal adenoma,” “adrenal mass,” “adrenal nodule,” “indeterminate adrenal,” and related keywords (Supplemental Table S1) to capture reports describing adrenal lesions.

### Study population

The first imaging examination in the study period in which an adrenal mass was reported was designated as the index study, regardless of whether the lesion was retrospectively visible on prior imaging. Patients with adrenal lesions smaller than 1 cm were excluded. Patients with previously known adrenal masses, defined as lesions clinically recognized for more than 30 days prior to the index imaging study, were also excluded.

### Imaging review

All prior cross-sectional imaging studies and the most recent available cross-sectional imaging up to one year after the study period (through December 31, 2022) were reviewed by the study radiologists. For patients with indeterminate diagnoses, additional imaging review was performed for all available radiologic studies through November 1, 2025. Radiologic image review was performed by one of three board-certified, fellowship-trained abdominal radiologists (MI, DRL, MJH). Prior to independent review, the radiologists evaluated 20 studies in consensus to standardize imaging assessment and data collection procedures. Radiologists were blinded to clinical outcomes during imaging review. For each lesion, imaging features recorded included laterality, maximum axial diameter, minimum axial diameter, presence of extra-adrenal extension, macroscopic fat, calcification, cystic change, or hemorrhage. Lesion roundness was calculated as the ratio of the maximum to minimum axial diameter.

### Imaging context and clinical indication

For the index imaging study, the clinical setting (outpatient, inpatient, or emergency department) and the clinical indication for imaging were recorded. Clinical indications were categorized to identify examinations performed for cancer staging or restaging, screening in patients with genetic predisposition syndromes, evaluation of hormone excess, or evaluation of suspected adrenal insufficiency. Adrenal incidentalomas were defined as adrenal masses ≥ 1 cm detected on imaging performed for indications unrelated to suspected adrenal pathology or active cancer staging/restaging in patients with prior malignancy or known genetic predisposition syndrome.

### Radiology report analysis

Radiology reports for the index imaging study were systematically reviewed to evaluate reporting practices related to adrenal lesions. The primary outcome variable was whether the adrenal lesion described in the report body (findings section) was included in the radiology report impression. For each report, the presence of the adrenal lesion in the impression, lesion characterization or diagnostic terminology used by the radiologist, descriptive imaging features documented in the findings section, and the presence or absence of management recommendations were recorded. When recommendations were provided, the suggested imaging modality and timing of follow-up were also recorded. Reports were reviewed independently by the study radiologists, and discrepancies were resolved by consensus review.

### Clinical data and follow-up

Demographic and clinical data were extracted from the electronic medical record using a data brokerage system supported by the Informatics Core Services at Washington University in St. Louis. Clinical records were reviewed for the 12-month period following the index imaging study to determine whether patients underwent biochemical testing for adrenal hormonal activity, evaluation by an endocrine specialist (endocrinology or endocrine surgery), biopsy, and/or adrenalectomy. Laboratory and clinical data were reviewed by a board-certified endocrinologist (NG) to determine whether hormonal evaluation was complete. Complete hormonal evaluation was defined as performance of a dexamethasone suppression test in all patients with adrenal masses, screening for primary aldosteronism in patients with hypertension, and measurement of plasma or urinary fractionated metanephrines in patients with indeterminate or lipid-poor adrenal lesions.

Final lesion classification was determined using imaging findings, biochemical results, and histopathologic data when available. Lesions were categorized as nonfunctioning adenoma, adenoma with unclear hormonal status, functioning adenoma (including aldosteronoma, mild autonomous cortisol secretion, Cushing syndrome, or androgen-producing adenoma), myelolipoma, pheochromocytoma, adrenal cortical carcinoma, adrenal metastasis, cyst, hematoma, or other diagnosis. Imaging diagnostic criteria used for lesion classification are summarized in Table 1.

**Table 1:** Imaging diagnostic criteria of benign adrenal lesions

Diagnosis	Imaging criteria
Adenoma	- HU < 10 and no primary malignancy containing microscopic fat e.g. hepatocellular or renal cell carcinoma, OR - HU < 20 with no significant growth over 5 years (< 1 mm/year), and no known malignancy or predisposition syndrome, OR, - Characteristic washout features with negative workup for pheochromocytoma - Signal loss on opposed phase imaging and no microscopic fat-containing primary malignancy (e.g. HCC, RCC)
Hematoma	- High density on non-contrast imaging, and no-enhancement on portal venous phase; OR, - Resolution within 6 months without any treatment
Myelolipoma	- Measurable region of interest with ≤ − 30 HU
Cyst	- Low density lesion with no measurable enhancement (< 10 HU) on a multiphase study

### Statistical analysis

Statistical analysis was performed using RStudio (RStudio version 2025.09.2). Descriptive statistics were calculated for demographic, imaging, and reporting variables. Continuous variables were compared using Student’s t-test or Welch’s t-test depending on variance equality, and nonparametric comparisons were performed using the Mann–Whitney U test. Categorical variables were compared using chi-square tests. Logistic regression analysis was performed to identify predictors of adrenal lesion inclusion in the report impression, including lesion size, age, prior malignancy, and mechanism of discovery. Odds ratios with 95% confidence intervals were reported, and a p-value less than 0.05 was considered statistically significant.

## Results

### Study cohort

**Fig. 1:**
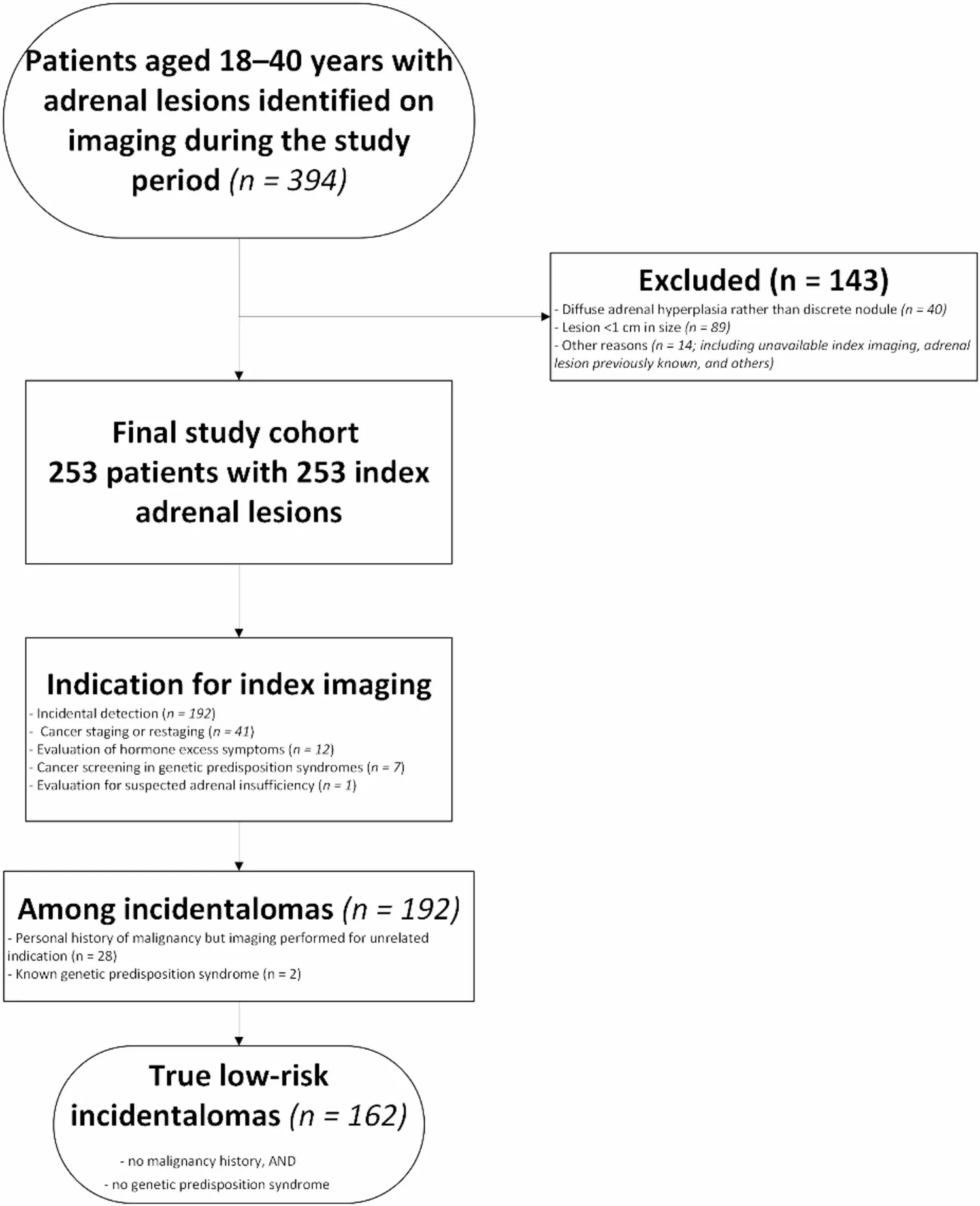
Flowchart of study cohort

A total of 253 patients with 253 index adrenal lesions met inclusion criteria (Fig. 1). The primary analyses of radiology reporting practices were performed using the full cohort of 253 adrenal lesions. The cohort included 142 (56.1%) women, and 154 (60.9%) white patients, with a mean age of 34.1 years (SD 5.5; range, 18.6–40.96). Among these, 41 examinations were performed for cancer staging or restaging (16.2%), 12 for evaluation of hormone excess symptoms (4.7%), 7 for screening in patients with genetic predisposition syndromes (2.8%), and one for evaluation of suspected adrenal insufficiency (0.4%). Of the remaining 192 lesions, 28 patients had a personal history of malignancy and two had a known genetic predisposition syndromes, resulting in a low-risk incidentaloma cohort of 162 patients. Secondary subgroup analyses were performed in the lower-risk incidentaloma subgroup (*n* = 162) to better characterize lesion outcomes in a clinically homogeneous population.

### Classification of adrenal lesions

Across the entire cohort of 253 lesions, classification was established by surgical pathology in 27 patients, biopsy in 2 patients, and imaging follow-up in 160 patients, with 137/253 (54.2%) benign lesions, 40/253 (15.8%) malignant lesions, and 64 lesions remaining non-characterized (Table 2).

**Fig. 2:**
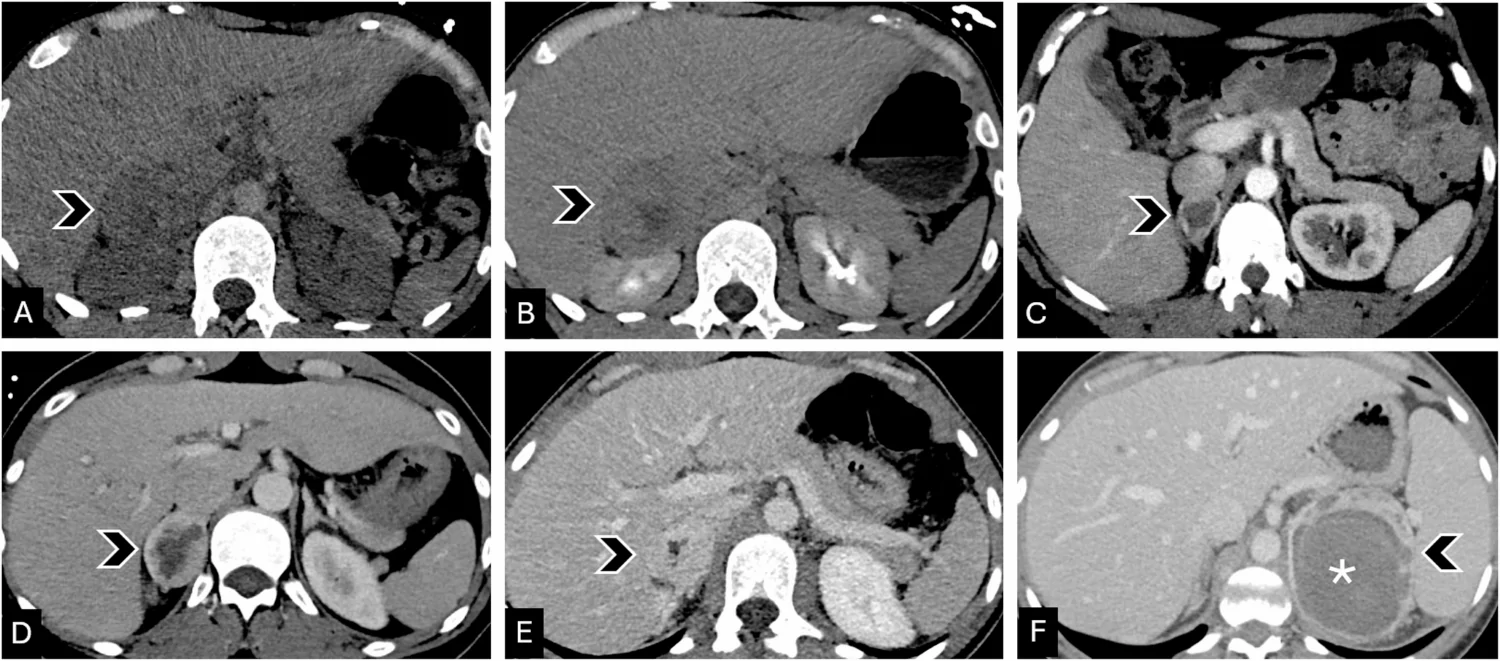
Index imaging features of five incidental pheochromocytomas (arrowheads). **A**, **B** A 4.1 cm mass detected on (**A**) early arterial phase CT chest performed for pulmonary embolism, with CT density of 35HU. **B** Internal heterogeneity in the same lesion as A is better appreciated on a delayed acquisition performed upon detection. **C**–**E** Adrenal masses in 3 different patients, measuring 3.1 cm, 4.4 cm, and 3.9 cm respectively, all demonstrate brisk rim hyperenhancement with internal heterogeneity. **F** A rim-enhancing heterogeneous 8.9 cm adrenal mass with a large internal hemorrhage (asterisk) in a patient with acute left flank pain

In the low-risk population of 162 incidentalomas, there were no malignancies: 5/162 (3.1%) were pheochromocytomas, 115/162 (71.0%) were benign masses, and 42/162 (25.9%) remained non-characterized. Of the five pheochromocytomas, two patients were subsequently found to have previously undiagnosed genetic predisposition syndromes (one with von Hippel–Lindau and one with multiple endocrine neoplasia type 2). Clinical presentation included hemorrhage with flank pain in one patient, incidental detection during CT performed for gastrointestinal symptoms or abdominal pain in two patients, and evaluation of dyspnea and palpitations in two patients (Fig. 2).

**Table 2:**
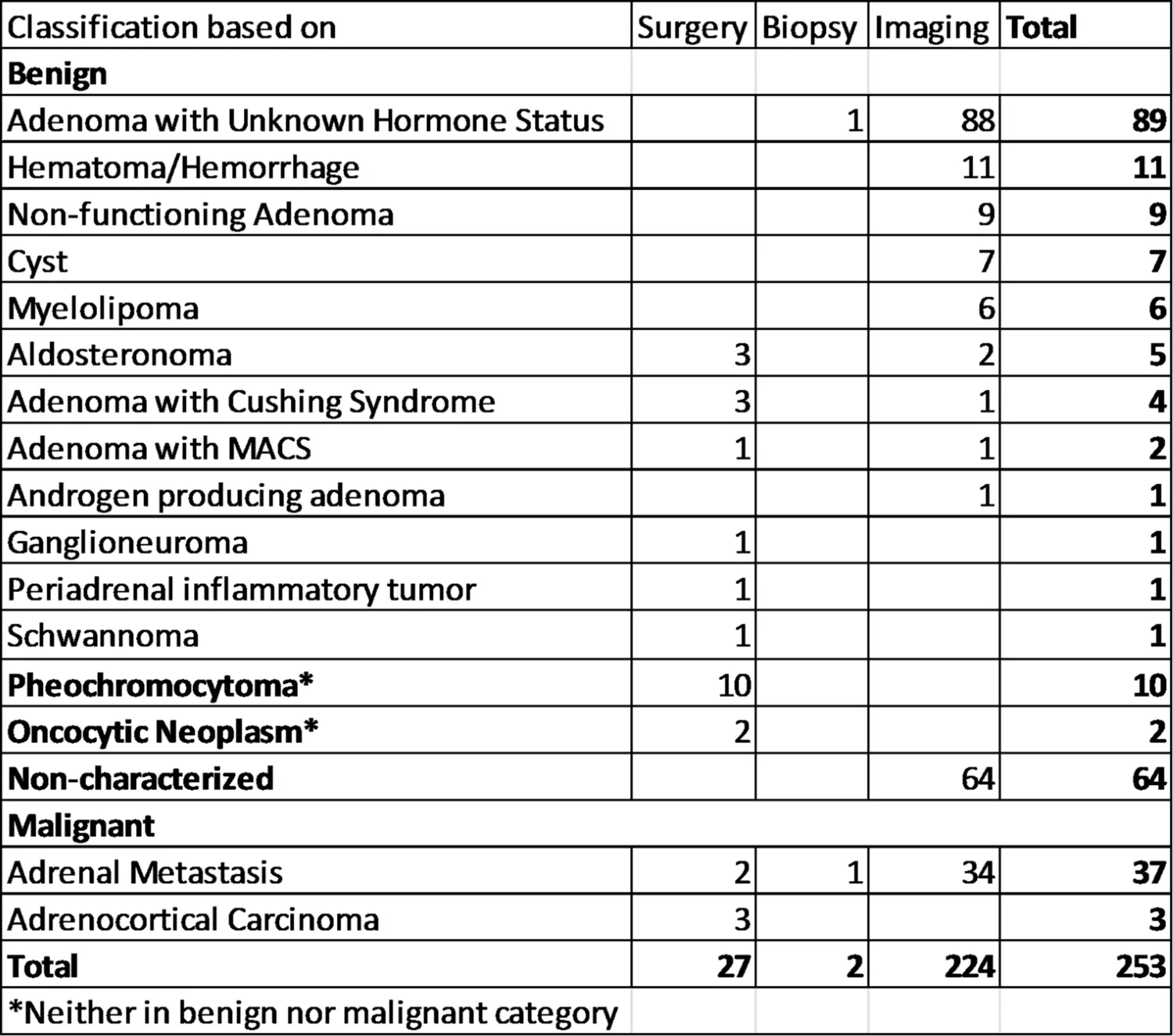
Basis for classification of adrenal lesions

### Imaging characteristics of adrenal lesions

Among the 253 index adrenal lesions, the index imaging modality was CT in 225 patients (172 contrast-enhanced, 30 non-contrast, and 23 without and with contrast), MRI in 23 patients (20 without and with contrast and 3 without contrast), and FDG PET/CT in 5 patients. Index lesions were left-sided in 144, right-sided in 90, and bilateral in 19. Mean lesion diameter was 2.37 cm (SD 1.6; range 1–16.6 cm). Lesions were slightly larger on the right (mean 2.61 cm) than on the left (mean 2.21 cm) (*p* = 0.046 by Welch t-test). Lesions on the left were significantly rounder than those on the right, with a mean aspect ratio (maximum/minimum axial diameter) of 1.30 vs. 1.41, respectively (*p* = 0.009).

### Radiology report content

Reports of the entire cohort of 253 lesions were analyzed based on content of the findings (body) and the impression sections of the radiologic report. Within the report body, lesion size was the most commonly reported descriptor (211/253 reports, 83.7%), followed by radiologist characterization such as indeterminate (71, 28.1%) or consistent with an adenoma (66, 26.1%). Hounsfield unit measurements were present in only 36 reports (14.3%).

Within the report impression, the adrenal lesion was mentioned in 194/253 (76.7%) of reports and lesion size was reported in the impression in 80/253 cases (31.6%). This was similar for the cohort of 162 incidentalomas where the lesion was mentioned in the impression in 121/162 (74.7%) cases and the lesion size was reported in the impression in 53/162 (32.7%) incidentalomas. Frequencies of specific descriptors in the findings and impression sections are summarized in Table 3.

**Table 3:**
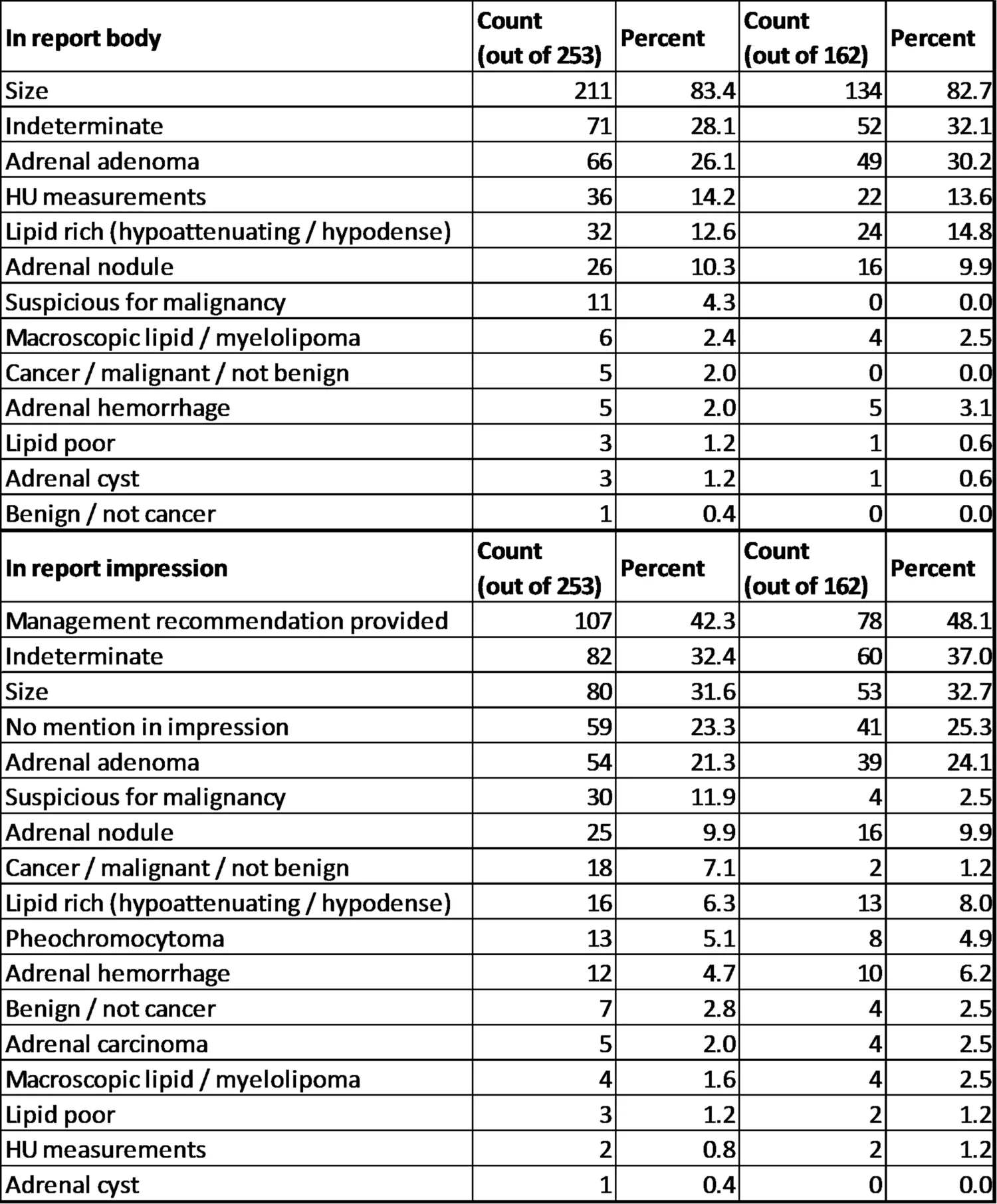
Frequency of descriptors mentioned in the report findings and impression sections

### Management recommendations

For the entire cohort, 146/253 reports (57.9%) contained no management recommendation in the impression. When recommendations were provided, interval imaging follow-up was most common (99/253, 39.2%). Hormonal evaluation was recommended in 7 cases (2.8%), biopsy was recommended in 3 cases (1.2%), and referral to an endocrine specialist in 2 cases (0.8%). Recommended imaging, when specified, was most frequently an abdominal MRI (69/85, 81.2%), followed by adrenal protocol CT (46/85, 54.1%). Specific follow-up timing was provided in 23/253 cases (9.1%). This was comparable for the 162 incidentalomas, where around half (78/162, 48.1%) had follow-up recommendation provided, most commonly for subsequent imaging [74/162 cases (45.7%) compared to 99/253 (39.2%), χ² = 1.74, *p* = 0.19].

### Predictors of inclusion in the impression

For the entire cohort, maximum lesion diameter was significantly larger when the lesion was explicitly included in the impression (median 2.1 cm, mean 2.49 ± 1.73 cm) compared with cases where it was omitted (median 1.7 cm, mean 1.96 ± 1.01 cm; Welch t = 2.93, *p* = 0.0039). This association remained significant using non-parametric testing (Mann–Whitney U = 7121, *p* = 0.0045). This also persisted for the incidentaloma group (*n* = 162) where inclusion in the impression was associated with larger lesion size (median 2.1 vs. 1.6 cm, mean = 2.50 ± 1.46 vs. 1.86 ± 0.82 cm, Welch t = 3.47, *p* = 0.00072, Mann–Whitney U = 3354, *p* = 0.00076).

Logistic regression demonstrated that each 1 cm increase in lesion size was associated with increased odds of inclusion in the impression (OR 1.45, 95% CI 1.05–2.01, *p* = 0.024). On multivariable logistic regression adjusting for age, lesion size, prior malignancy, and mechanism of discovery, lesion size remained independently associated with inclusion in the impression (OR 1.46 per cm increase; 95% CI 1.05–2.02; *p* = 0.025). Age, prior malignancy, and mechanism of discovery were not independently associated with impression inclusion.

Adrenal lesions detected during cancer staging were omitted from the impression in 24.4% of reports (10/41) compared with 17.9% for other indications (38/212) (χ² = 0.94, *p* = 0.33). Lesions discovered during evaluation of hormone excess symptoms were omitted from the impression in 18.2% (2/11) of cases, similar to other mechanisms of discovery (19.0%, 46/242; χ² = 0.005, *p* = 0.94).

Inclusion of the adrenal lesion in the impression was not associated with imaging context (emergency department, inpatient, outpatient, or unknown) (Supplemental Table S2).

### Clinical follow-up and endocrine evaluation

Among the entire 253 adrenal lesion cohort, 57 patients were ultimately evaluated by an endocrine specialist. Endocrine or endocrine surgery evaluation occurred more frequently when the adrenal lesion was included in the report impression (25.3% vs. 13.6%; OR 2.16, 95% CI 0.96–4.85; *p* = 0.06), although this did not reach statistical significance. Hormonal workup was complete in 22/253 (8.7%) patients, partial in 34 (13.4%) patients, and not performed in 197 (77.9%) cases. Out of 22 patients with complete hormonal workup, 5 had functional adenomas (3 with Cushing and 2 with MACS), 9 had non-functional adenomas, and the remaining 8 had variable other diagnoses (2 adrenocortical carcinoma, 2 cysts, one pheochromocytoma, one myelolipoma, one schwannoma, and one oncocytic neoplasm). In 3 patients where a biopsy was recommended, none had a biopsy. Two went on to surgery, with surgical pathology consistent with adrenocortical carcinoma in one patient, and an oncocytic neoplasm in the other. The third biopsy recommendation was a suggested site in a patient with diffuse metastases from an undifferentiated sarcoma that was eventually diagnosed after a liver biopsy.

Lesions were classified as benign, malignant, pheochromocytoma, or oncocytic neoplasms in 189 patients (74.7%) of the entire cohort, although a large number of lesions remained as adenomas of unclear hormonal status due to lack of hormonal testing. The odds of reaching a specific final diagnosis (e.g. aldosteronoma or schwannoma, compared to nonfunctional adenoma or non-characterized pathology) was significantly higher when the adrenal lesion was included in the report impression (88/194 [45.4%] vs. 12/59 [20.3%]; OR 3.25, 95% CI 1.65–6.41; *p* < 0.001) as detailed in Table 4.

**Table 4:**
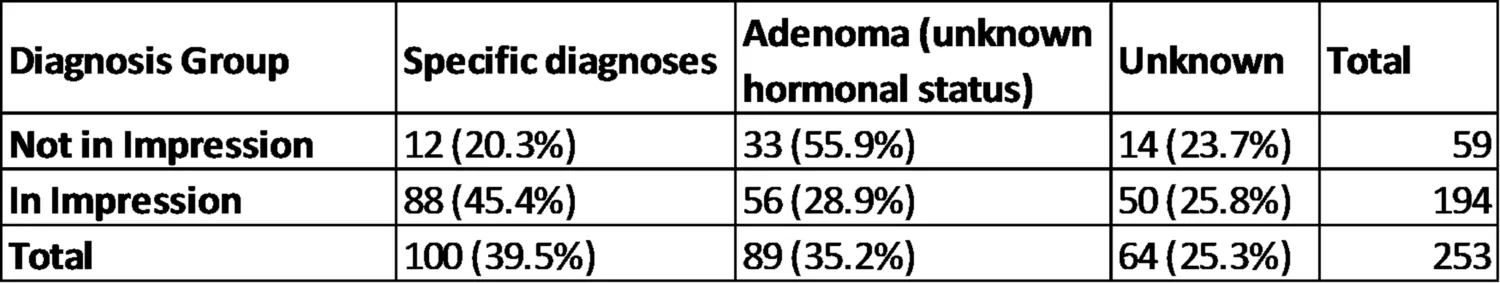
Specific diagnoses reached as related to presence of the adrenal mass in the impression

## Discussion

In this study of adrenal lesions detected on cross-sectional imaging in young adults, we observed several important patterns in radiology reporting and downstream clinical evaluation. First, nearly one quarter of adrenal lesions described in the report body were not included in the impression, and inclusion in report impression appeared to be driven primarily by size rather than the clinical context or indication for imaging. Second, key imaging descriptors were frequently omitted, with quantitative attenuation measurements reported in only a minority of reports. Third, the odds of reaching a specific diagnosis was higher when the adrenal mass was included in the impression section of the report, although this association may partly reflect confounding by lesion size or imaging features that independently prompted further evaluation. Finally, management recommendations were inconsistent, with more than half of reports lacking any guidance regarding follow-up imaging or biochemical evaluation. Together, these findings highlight substantial variability in adrenal lesion reporting and suggest opportunities to improve the consistency and clinical utility of radiology reports for adrenal incidentalomas.

Radiology report language plays an important role in guiding downstream clinical care. The impression section of a radiology report typically serves as the primary communication point for referring clinicians and often drives subsequent diagnostic and management decisions. Findings described only in the body of a report may receive less clinical attention than those emphasized in the impression, particularly in busy clinical settings where the impression is frequently the only portion of the report reviewed in detail.

Our findings showed that endocrine specialty referral and hormonal evaluation recommendations occurred in a minority of cases. These findings highlight the need to educate radiologists about the importance of hormonal workup in young adults with adrenal incidentalomas. Prior studies have demonstrated that radiology report structure and language can influence downstream care. Corwin et al. reported that structured reporting and standardized recommendations improved adherence to guideline-based evaluation of adrenal incidentalomas [18]. Similarly, De Haan et al. showed that patients referred to endocrine specialists were significantly more likely to undergo appropriate biochemical testing and diagnostic workup [9].

Among our cohort of adrenal lesions, 5/14 (35.7%) of those with adenomas who had complete hormonal workup demonstrated evidence of hormonal hypersecretion; this value may be exaggerated due to selection bias although our rate of 64.3% nonfunctional adenomas aligns with the wide range of 40–70% reported in the ENSAT guideline [7]. Early identification and treatment of hormonally active adrenal tumors is of particular importance in younger patients, in whom untreated endocrine disorders may contribute to long-term complications including hypertension, cardiovascular disease, and metabolic dysfunction [19, 20]. Despite evidence that early management and detection may be pertinent in younger adults, hormonal evaluation was recommended in only a small minority of radiology reports in our cohort. The aforementioned study by Ebbehoj et al. found only about 10% of nearly 1300 patients had sufficient workup within 6 months of adrenal mass detection [16]. These findings suggest that functional evaluation of adrenal lesions may be underemphasized in routine radiology reporting.

The variability in reporting observed in this study highlights the absence of widely adopted standardized reporting frameworks for adrenal lesions. In contrast to other common imaging findings, such as breast, thyroid, and liver lesions, where structured reporting systems such as BI-RADS, TI-RADS, and LI-RADS, respectively, have improved communication and management consistency, adrenal lesions currently lack a broadly implemented reporting standard. Several groups have proposed structured reporting approaches or decision-support tools for adrenal incidentalomas, which have been associated with improved guideline adherence and more consistent follow-up recommendations [9, 21]. Whereas there is potential confounding by lesion size or features that render more concerning lesions more likely to both be included in the impression and undergo additional workup, our findings highlight the variability in current radiology reporting practices, and support the potential value of including adrenal lesions in the impression, and explicit management recommendations when appropriate. Such approaches may improve communication between radiologists and referring clinicians while helping to balance appropriate evaluation with avoidance of unnecessary testing.

Another notable finding of this study is that inclusion of adrenal lesions in the report impression was strongly associated with lesion size but not with imaging context or clinical indication for the imaging study. This suggests that radiologists may rely primarily on lesion size when determining whether an adrenal finding warrants emphasis in the impression. Although lesion size is an important predictor of malignancy risk in adrenal lesions, reliance on size alone may overlook clinically relevant features such as functional potential or patient-specific risk factors. For example, hormonally active adrenal tumors may be relatively small yet clinically significant, particularly in younger individuals. The absence of an association between imaging context and impression inclusion in our cohort further suggests that clinical information available at the time of imaging interpretation may not consistently influence reporting emphasis for adrenal lesions.

The clinical context of imaging also raises additional considerations for adrenal lesions detected during cancer staging examinations. In such settings, adrenal lesions may represent metastatic disease, although benign adrenal nodules are also common in patients with malignancy. In our cohort, adrenal lesions detected during cancer staging had similar likelihood to be omitted from the impression than those discovered in other clinical contexts. However, the clinical implications of adrenal lesions in oncologic imaging are complex and may depend on factors such as the primary malignancy type, imaging appearance, and presence of additional metastatic disease. In our cohort, biopsy was recommended in 3 patients, but none of them underwent a biopsy. It is important for radiologists to realize that the decision to biopsy an adrenal mass is often more complex than other organ masses and that referral to an endocrine specialist is usually more appropriate than recommending a biopsy. Additionally, in patients with malignancy, there may be a propensity to biopsy suspicious adrenal masses for cancer staging purposes; however, even among patients with prior malignancy, there is still a risk for pheochromocytoma and metanephrine evaluation must be completed prior to sampling any indeterminate adrenal mass [7, 22]. There are also clinical scenarios, such as patients with high suspicion for adrenocortical carcinoma, where biopsy is superfluous to their clinical course and has theoretical risks of tissue seeding. These findings underscore the importance of clear radiology reporting to guide appropriate evaluation of adrenal findings in both oncologic and non-oncologic settings. From an endocrine specialist standpoint, inclusion of certain adrenal mass descriptors in the radiology report can be helpful to improve clinician communication regarding adrenal nodules, namely: size, homogeneity, Hounsfield unit measurements on various phases to reflect presence, degree, and pattern of enhancement, presence and estimated proportion of macroscopic or microscopic fat, invasion of adjacent structures, and stability as compared to prior studies when applicable.

This study has several limitations. First, the retrospective design and keyword-based radiology search may introduce selection bias and limit the ability to establish causal relationships between reporting practices and downstream clinical outcomes. Second, although imaging review and report abstraction were performed by experienced abdominal radiologists, interpretation of radiology report language may involve some degree of subjectivity. Third, the study was conducted at a single academic institution, and reporting practices may differ across institutions or practice settings. Additionally, we did not adjust for clustering by individual radiologists because the number of radiologists was large relative to the sample size, limiting the feasibility and interpretability of radiologist-level mixed-effects modeling. Because cases were identified through report text, the study may have missed adrenal lesions that were not described using the selected terms, were described nonspecifically, or were omitted from the report. Associations between impression inclusion and downstream clinical evaluation may be confounded by lesion size, imaging appearance, or perceived clinical suspicion, as more suspicious lesions may have been both more likely to be emphasized in the report impression and more likely to undergo additional workup. Moreover, although extended imaging follow-up was available for many patients, a subset of adrenal lesions remained non-characterized, which may affect estimates of lesion prevalence and outcomes. Finally, our classification of benign lesions relied in part on conservative imaging stability criteria, which may differ from approaches used in other studies.

In conclusion, adrenal lesions detected on cross-sectional imaging in young adults are frequently omitted from radiology report impressions and often lack key imaging descriptors or management recommendations. Radiology reporting practices in this cohort appeared to be influenced primarily by lesion size rather than clinical context. These findings highlight opportunities to improve the clarity and clinical impact of adrenal lesion reporting, particularly in younger patients in whom functional tumors may carry important long-term health implications.

## Supplementary Information

Below is the link to the electronic supplementary material.

Supplementary Material 1

## Data Availability

No datasets were generated or analysed during the current study.
