# Supplementary material for: Adrenal lesions in young adults: variability in radiology reporting and implications for clinical evaluation
Source: Abdom Radiol (NY). 2026 Jun 19;51(12):6620–30. doi: 10.1007/s00261-026-05606-x (PMC13768372; doi:10.1007/s00261-026-05606-x)
Supplement: Supplementary file 1 [file 261_2026_5606_MOESM1_ESM.docx]

**Supplemental Tables:**

Table S1: List of terms for radiology information system search

**Terms**

Incidentaloma

Adrenal mass

Adrenal nodule

Adrenal adenoma

Adrenal thickening

Adrenal nodularity

Adrenal hyperplasia

Adrenal indeterminate

Indeterminate adrenal

Adrenal lipid rich

Adrenal lipid-rich

Adrenal lipid poor

Adrenal lipid-poor

lipid rich adrenal

lipid-rich adrenal

lipid poor adrenal

lipid-poor adrenal

Table S2: Inclusion of Adrenal Mass in Report Impression by Clinical Context
